# Optimization of biogenic silver particle synthesis for methylene blue degradation

**DOI:** 10.1098/rsos.250402

**Published:** 2025-08-27

**Authors:** Gülçin Demirel Bayik, Busenur Baykal

**Affiliations:** ^1^ Department of Environmental Engineering, Zonguldak Bulent Ecevit Universitesi, Zonguldak, Turkey

**Keywords:** sustainable synthesis, factorial design, silver particles, green synthesis, dye degradation

## Abstract

This study presents an optimization of the sustainable synthesis of silver particles (AgPs) derived from hazelnut leaves employing a full factorial design. Four synthesis parameters were systematically evaluated at two levels: the water-to-leaf ratio (LW), extract-to-AgNO₃ ratio (EAg), AgNO₃ molarity (Mol), and plant leaf size (LS). Statistical analysis revealed that LW and the interaction between EAg and Mol are significant factors influencing the synthesis yield of AgPs. In contrast, Mol, LS and the EAg × Mol interaction were determined to be the key factors affecting the efficiency of dye degradation. The optimized AgPs demonstrated enhanced degradation kinetics, following a pseudo-second-order model (*k*_2_ = 67 × 10⁻³ mg g⁻¹ min⁻¹, *R*² = 0.99) and fitting well with Langmuir–Hinshelwood kinetics (*k*_app_ = 5.9 min⁻¹, *R*² = 0.88). Scanning electron microscopy with energy-dispersive X-ray (EDX) analysis and particle size analysis confirmed that AgPs optimized for dye degradation possessed smaller particle sizes and larger surface areas (0.201 m² g^−1^ versus 0.113 m² g^−1^), which contributed to improved catalytic performance. EDX analysis revealed a higher carbon and oxygen content in these AgPs, indicating the presence of surface functional groups that promote adsorption. Although the overall degradation efficiency of AgPs was slightly lower than that of certain other nanoparticle systems, their kinetic performance was comparable. This study emphasizes the critical role of synthesis optimization in enhancing catalytic activity and highlights AgPs as a promising eco-friendly catalyst for wastewater treatment applications.

## 1. Introduction

Green synthesis approaches utilizing plant extracts for the fabrication of metal nanoparticles (NPs) or microparticles with photocatalytic applications are gaining increased attention due to their simplicity and high efficiency. Unlike physicochemical methods, green synthesis does not produce harmful compounds or by-products [1]. Compared to conventional methods, the green synthesis of metal or microparticles offers multiple advantages: (i) biocompatibility, (ii) method simplicity, (iii) utilization of natural resources (plants, fungi, algae and microorganisms), and (iv) non-toxicity, making the resulting particles appropriate for a wide range of applications. Additionally, (v) the process eliminates the need for external capping or stabilizing agents, (vi) is cost-effective owing to minimal or no energy requirements, (vii) is amenable to large-scale production, (viii) ensures reproducibility and (ix) the synthesized particles have a well-defined morphology [2,3].

Applications for micro- and nanostructured systems are developing including delivery, biomarkers, surface-enhanced Raman scattering sensors, catalysis and antimicrobial activity [4]. Owing to their unique physicochemical properties, metal oxide NPs have demonstrated significant utility in chemistry and environmental science. Their high density and large specific surface have enabled notable outcomes, particularly in innovative adsorbent-based applications. Among available techniques, adsorption stands out as a simple, cost-effective, reliable, and robust separation method that has been extensively employed for the remediation of contaminated water. This approach has consistently proven to be effective in the removal of water impurities for a number of years [5]. The functionality of metal particles for a range of applications can be significantly enhanced by adjusting their size and shape. Morphological characteristics can be precisely controlled by manipulating various experimental parameters, including reaction duration, reactant concentration, pH, temperature, aeration, salt content, etc. In the context of biologically mediated synthesis of metal NPs, stringent control over these variables is often critical for optimizing structural and functional outcomes [6]. A key initial step in creating a synthesis route is optimizing the parameters. The simultaneous and sequential approaches are the names given to the standard systematic optimization processes [7]. The optimal values of each variable are determined once under specific conditions in a univariate design. The process requires a substantial amount of time and money to optimize experimental conditions by adjusting one variable while maintaining all others at a fixed level. Furthermore, it is not appropriate for determining the interactions among multiple factors [8]. In such methods, the response surface is explored incrementally—typically through stepwise optimization—until an optimal solution is identified. However, these methods often suffer from significant limitations, including the complexity of navigating multidimensional response surfaces and their inherently slower convergence. In contrast, these challenges are largely mitigated by simultaneous optimization techniques, such as factorial and mixture designs, which are more effective in evaluating factor interactions comprehensively [7]. The experimental trials in a factorial design are carried out according to a pre-established plan. Once the experimental data are collected, optimal conditions are identified using techniques such as response surface analysis or retention mapping [7]. Among the most widely used approaches are two-level (2k) factorial designs, in which responses are measured at every possible combination of factor levels. This full factorial structure allows for the simultaneous evaluation of the individual and interactive effects of multiple parameters, providing a comprehensive understanding of their influence on process optimization [9].

In recent years, numerous studies have investigated the green synthesis of metal NPs using various plant extracts, often in combination with experimental design methodologies to optimize synthesis conditions. For example, Kartini *et al.* synthesized silver NPs using extracts from *Phyllanthus niruri* (PN), *Orthosiphon stamineus* (OS) and *Curcuma longa* (CL) at different concentrations, identifying 0.5% PN extract as the most effective based on particle yield and characteristics [10]. Similarly, factorial and response surface methodologies have been widely employed to evaluate the influence of synthesis parameters. In one study, NaOH concentration emerged as the dominant factor affecting silver NP synthesis using oregano extract [11]. Another study applied central composite design (CCD) to optimize the biosynthyesis of silver NPs from banana peel extract (BPE), highlighting the critical roles of pH, precursor concentration and incubation time on NP quality and stability [12]. Comparable optimization techniques have also been applied to other metal oxide NPs such as copper oxide (CuO) and zinc oxide (ZnO), where factors including extract volume, metal salt concentration, reaction temperature and reaction time significantly influenced particle size, morphology and surface properties [13,14].

In this study, a two-factorial experimental design was employed to optimize the synthesis of silver particles (AgPs) using hazelnut leaf extract. The effect of four key factors—leaf-to-water (LW) ratio, extract-to-AgNO_3_ (EAg) ratio, molarity of AgNO_3_ (Mol) and leaf size (LS)—were systematically investigated. The influence of both main effects and their interactions was evaluated for two response variables: particle synthesis yield and methylene blue (MB) degradation efficiency. Additionally, reaction kinetics were calculated to better analyse the mechanisms driving the degradation process.

## 2. Material and methods

### 2.1. Experimental design

**Figure 1.**
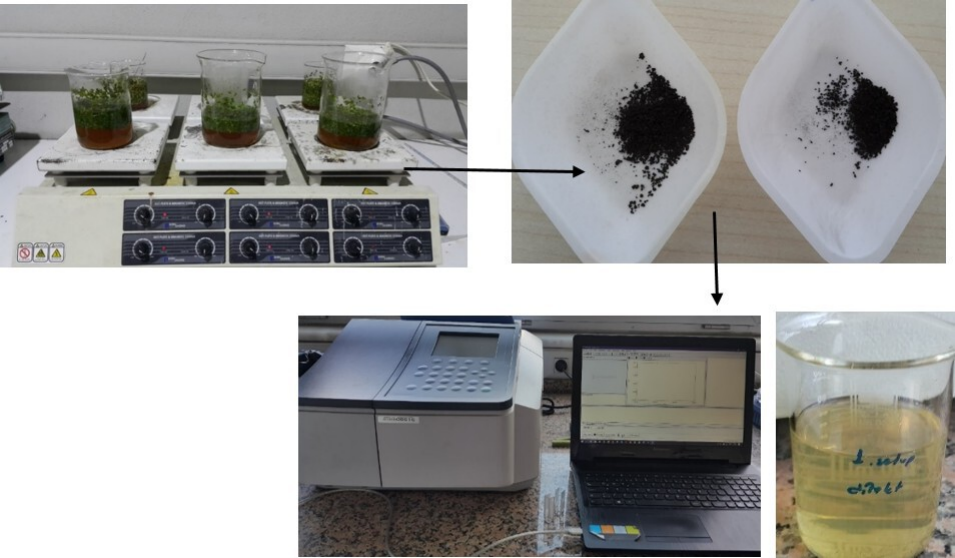
Experimental set-up.

The effects of four distinct parameters on the synthesis yield and MB degradation efficiency of AgPs were investigated using a full factorial experimental design with three replicates. The design and statistical analysis were performed using Minitab 18 software. Four independent factors, each at two levels, were selected for the study: the LW ratio during extraction, the EAg ratio, the Mol of solution, and the plant LS. The response variables were defined as the mass of synthesized AgPs (g) and dye removal efficiency. In total, 48 experiments were conducted, including three replicates per condition. The low and high levels of each factor are presented in table 1, and the experimental set-up is presented in figure 1.

**Table 1.** Experimental design parameters.

parameter	unit	low level (−1)	high level (+1)
leaf:water (LW)	g ml^−1^	1:7	1:15
extract:AgNO_3_ solution (EAg)	ml ml^−1^	1:4	1:10
molarity AgNO_3_ (Mol)	Mol	1	5
leaf size (LS)	mm	<1	2
response			
NP amount produced	g		
dye removal	%		

### 2.2. Plant biomass and extraction procedure

Hazelnut leaves were collected from the gardens in Zonguldak, Turkey. The leaves were initially rinsed with tap water and followed by distilled water, to remove surface contaminants. They were then air-dried at room temperature. Once dried, they were chopped and sieved through an 18-mesh screen. The processed leaves were stored in sealed plastic bags until further use.

For the extraction process, 12 g of the dried hazelnut leaves was weighed and transferred into a 250 ml beaker, and 180 ml of distilled water was added to maintain the desired leaf-to-water ratio. The mixture was stirred using a magnetic stirrer at 100°C for 10−15 minutes until the solution’s colour changed from yellow to brown, indicating successful extraction. After cooling to room temperature, the solution was filtered using a vacuum filtration system to obtain the plant leaf extract.

### 2.3. Synthesis and characterization of Ag particles

The previously prepared plant extracts were reacted with a specified volume of AgNO_3_ solution in a beaker to achieve the desired EAg ratio. The formation of silver NPs was indicated by a visible colour change and monitored using a Schimadzu UV-1800 spectrophotometer over a wavelength range of 300 to 700 nm for a duration of 24 hours. Upon completion of the reaction, the resulting mixture was dried in an incubator at 105°C for 24 hours. The beaker was then rinsed with distilled water, and the contents were filtered through Whatman No. 1 filter paper using a vacuum pump. Filter papers containing the NPs were further dried in an incubator at 105°C for 2 hours. The morphology and elemental composition of the synthesized NPs were characterized using scanning electron microscopy (SEM) and energy-dispersive X-ray (EDX) analysis. Particle size distribution was determined using a Mastersizer laser diffraction particle size analyser.

### 2.4. Batch degradation test

Dye degradation experiments were conducted in batch reactors using 50 ml beakers containing MB at an initial concentration of 0.5 mg l^−1^. A fixed amount of AgPs (0.02 g) was added to each beaker, and the samples were exposed to natural sunlight for 24 hours. At the end of the exposure period, aliquots were collected, and absorbance measurements were performed at 664 nm using a UV–visible spectrophotometer. The MB degradation was calculated by


(2.1) $$q_{e}=(C_{o}-C_{t})\times V/W,$$

(2.2) $$\text{Degradation}\;(\%)=[(C_{o}-C_{t})/C_{o}]\times 100,$$

where *C*_o_ is the initial concentration (mg l^−1^), *C*_t_ is the concentration at time *t* (mg l^−1^), *V* is the volume (l) of solution and *W* is the weight of AgPs (g).

## 3. Results and discussion

**Figure 2.**
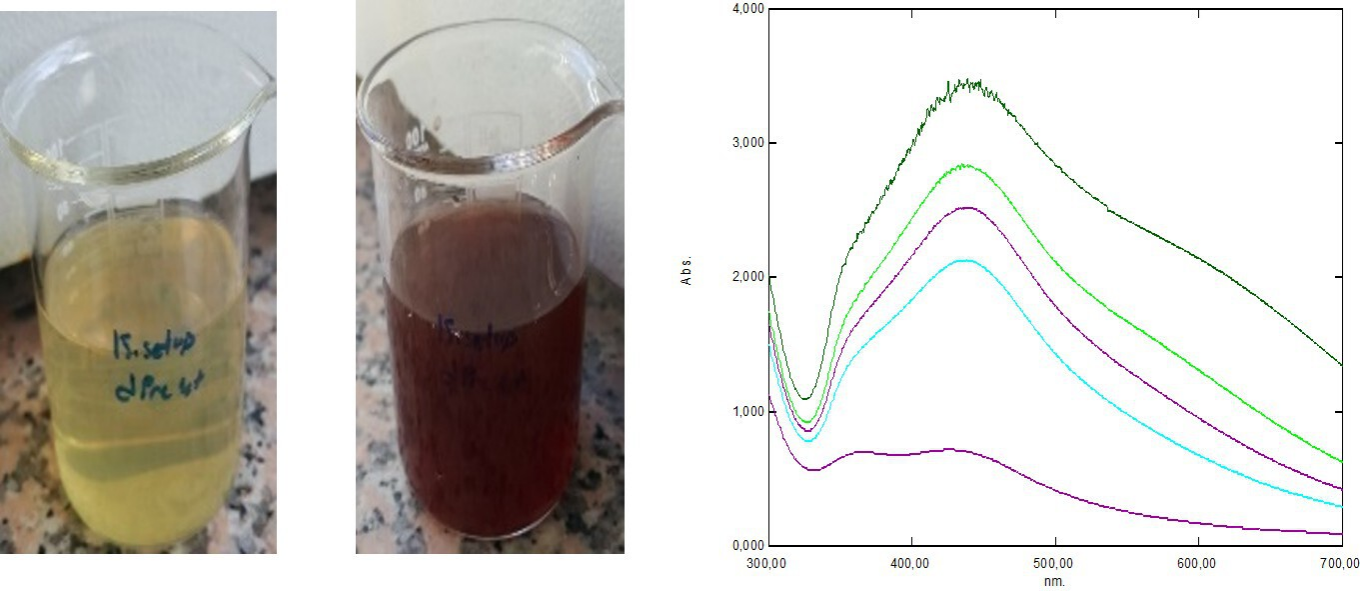
Colour change and increase in the UV absorption peaks during synthesis.

The formation of silver NPs was visually confirmed by a distinct colour change, as shown in figure 2. UV–visible spectrophotometric analysis revealed an increase in absorbance over time, particularly between 5 minutes and 24 hours, further indicating the progressive synthesis of NPs.

### 3.1. Analysis of the factorial design

A total of 48 experimental runs were conducted in triplicate, based on a full factorial design (FFD) involving four independent parameters each at two levels. These experiments were designed to evaluate both NP synthesis yield and MB degradation efficiency. The average values of each response are presented in table 2.

**Table 2.** Summary of the full factorial experimental design.

run	leaf:water (g ml^−1^)	extract:AgNO_3_ (ml ml^−1^)	Mol AgNO_3_ (mmol)	leaf size (mm)	response NP production (g)	response degradation (%)
1	1:7	1:4	1	1	0.1290	40.00
2	1:7	1:10	1	1	0.1533	62.85
3	1:15	1:10	1	1	0.084	42.40
4	1:15	1:4	5	1	0.2248	46.27
5	1:7	1:10	5	1	0.1192	37.03
6	1:7	1:4	1	2	0.0884	59.15
7	1:15	1:4	1	2	0.0894	53.53
8	1:7	1:10	1	2	0.1145	40.30
9	1:15	1:10	1	2	0.0735	47.40
10	1:7	1:4	5	2	0.1400	42.20
11	1:7	1:10	5	2	0.0700	36.53
12	1:15	1:10	5	2	0.1167	39.77
13	1:15	1:4	1	1	0.1600	45.60
14	1:15	1:4	5	2	0.1300	38.47
15	1:7	1:4	5	1	0.1700	31.40
16	1:7	1:4	1	2	0.0600	32.07

To determine the statistical significance of the model and the individual effects of each parameter, an ANOVA was performed. The results of the ANOVA for NP synthesis and dye degradation are provided in tables 3 and 4, respectively. In the ANOVA tables, d.f. represents the degrees of freedom, adj SS refers to the adjusted sum of squares, adj MS denotes the adjusted mean squares, *F* stands for the *F*-ratio (distribution factor), and *p* indicates the significance level. At a 95% confidence interval, a *p*-value less than 0.05 signifies that the corresponding factor is statistically significant. Among the evaluated factors, the one with the highest *F*-value is considered have the most significant influence.

**Table 3.** ANOVA for selected factorial model response of produced nanoparticles.

source	d.f.	adj SS	adj MS	*F*-value	*p‐*value	contribution (%)
model	15	0.066654	0.004444	2.89	0.008	
linear	4	0.044629	0.011157	7.26	0.000	
LW	1	0.042579	0.042579	27.71	0.000	39.43
EAg	1	0.000381	0.000381	0.25	0.622	0.35
Mol	1	0.002086	0.002086	1.36	0.254	1.93
LS	1	0.000191	0.000191	0.12	0.727	0.18
2-way interactions	6	0.014538	0.002423	1.58	0.192	
LW × EAg	1	0.000118	0.000118	0.08	0.783	0.11
LW × Mol	1	0.000807	0.000807	0.53	0.475	0.75
LW × LS	1	0.001248	0.001248	0.81	0.376	1.16
EAg × Mol	1	0.008402	0.008402	5.47	0.027	7.78
EAg × LS	1	0.000326	0.000326	0.21	0.649	0.30
Mol × LS	1	0.003521	0.003521	2.29	0.142	3.26
3-way interactions	4	0.000628	0.000157	0.10	0.981	
LW × EAg × Mol	1	0.000269	0.000269	0.18	0.679	0.25
LW × EAg × LS	1	0.000156	0.000156	0.10	0.752	0.14
LW × Mol × LS	1	0.000068	0.000068	0.04	0.835	0.06
EAg × Mol × LS	1	0.000159	0.000159	0.10	0.750	0.15
4-way interactions	1	0.000067	0.000067	0.04	0.836	
LW × EAg × Mol × LS	1	0.000067	0.000067	0.04	0.836	0.06
error	27	0.041493	0.001537			
total	42	0.108146				100

**Table 4.** ANOVA for selected factorial model response of dye degradation.

source	d.f.	adj SS	adj MS	*F*-value	*p‐*value	contribution (%)
model	15	2470.74	164.72	3.91	0.001	
linear	4	1617.09	404.27	9.61	0.000	
LW	1	0.38	0.38	0.01	0.925	0.010
EAg	1	194.37	194.37	4.62	0.040	5.33
Mol	1	1108.40	1108.40	26.34	0.000	30.38
LS	1	402.07	402.07	9.55	0.004	11.02
2-way interactions	6	830.21	138.37	3.29	0.014	
LW × EAg	1	0.26	0.26	0.01	0.938	0.01
LW × Mol	1	44.01	44.01	1.05	0.315	1.21
LW × LS	1	26.95	26.95	0.64	0.430	0.74
EAg × Mol	1	696.60	696.60	16.55	0.000	19.09
EAg × LS	1	30.30	30.30	0.72	0.403	0.83
Mol × LS	1	0.92	0.92	0.02	0.883	0.03
3-way interactions	4	196.27	49.07	1.17	0.347	0.00
LW × EAg × Mol	1	0.15	0.15	0.00	0.953	0.05
LW × EAg × LS	1	1.91	1.91	0.05	0.833	2.21
LW × Mol × LS	1	80.79	80.79	1.92	0.177	3.21
EAg × Mol × LS	1	117.19	117.19	2.78	0.106	1.05
4-way interactions	1	38.42	38.42	0.91	0.347	
LW × EAg × Mol × LS	1	38.42	38.42	0.91	0.347	1.05
error	28	1178.25	42.08			
total	43	3648.99				100

The ANOVA results confirm that the model provides a good fit to the experimental data (*p* < 0.05). For AgP synthesis, the LW and the two-way interaction between extract-to-AgNO₃ ratio and AgNO₃ molarity (EAg × Mol) were identified as statistically significant factors. None of the three-way interactions were found to be statistically significant. Among all variables, LW exhibited the highest contribution to AgP production (39.43%), followed by the EAg × Mol interaction (7.78%). The coefficient of determination (*R*²) for the model was 61.63%, with an adjusted *R*² value of 40.32%, indicating a moderate fit to the observed data.

For MB degradation, the parameters EAg, Mol and LS were statistically significant (*p* < 0.05), along with the two-way interaction EAg × Mol. Molarity had the greatest impact (30.38%), followed by LS (11.02%) and EAg (5.33%).

The *R*² value of the dye degradation model was 67.71%, and the adjusted *R*² was 50.41%, reflecting a reasonably strong explanatory power.

**Figure 3.**
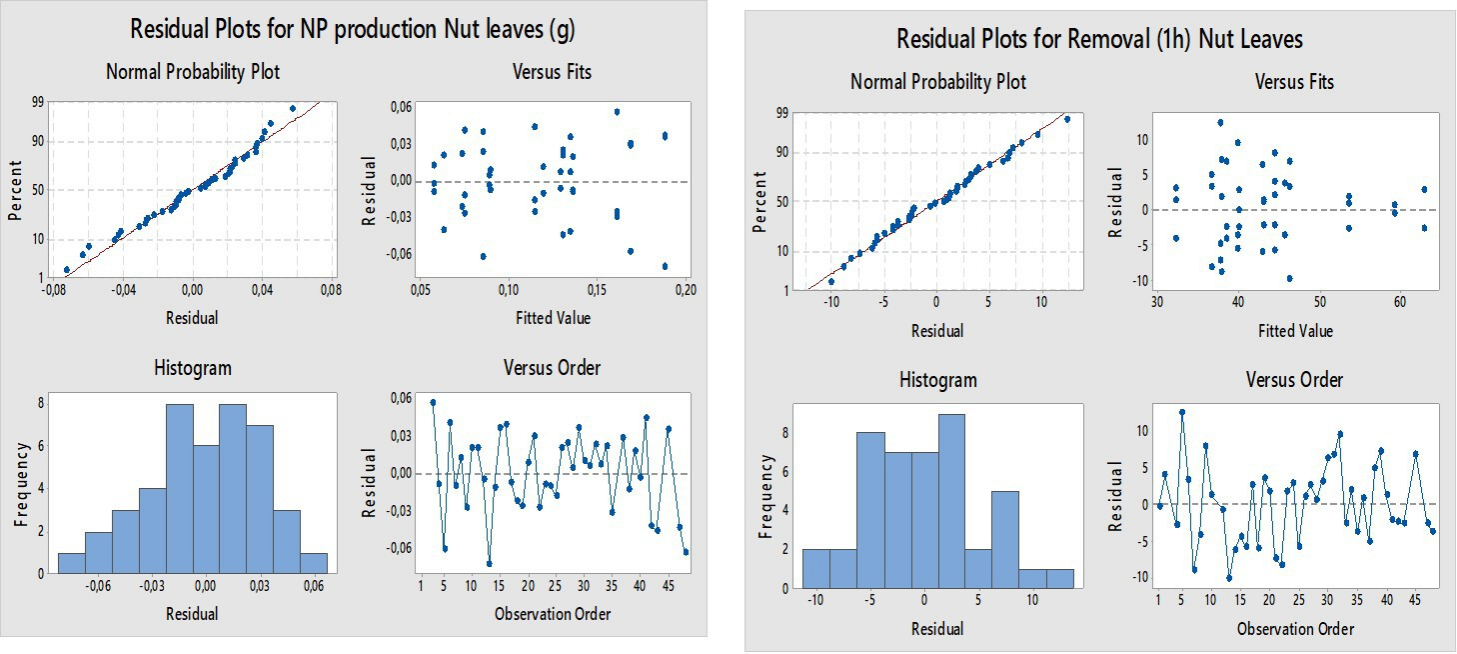
Analysis of residuals.

Figure 3 presents the residual analysis conducted to validate the assumptions underlying the ANOVA. The normal probability plot of the standardized residuals closely follows a straight line, suggesting that the residuals are approximately normally distributed with no significant deviations. Additionally, the plot of residuals versus fitted values shows no apparent pattern, indicating that the variance of the residuals is constant (homoscedasticity). The residuals are randomly scattered around zero, supporting the assumption of independence. Furthermore, the plot of residuals versus observation order shows no trend or systematic structure, confirming that there is no bias due to the time or sequence of data collection. Thus, the assumptions of normality, constant variance and independence were satisfactorily met.

**Figure 4.**
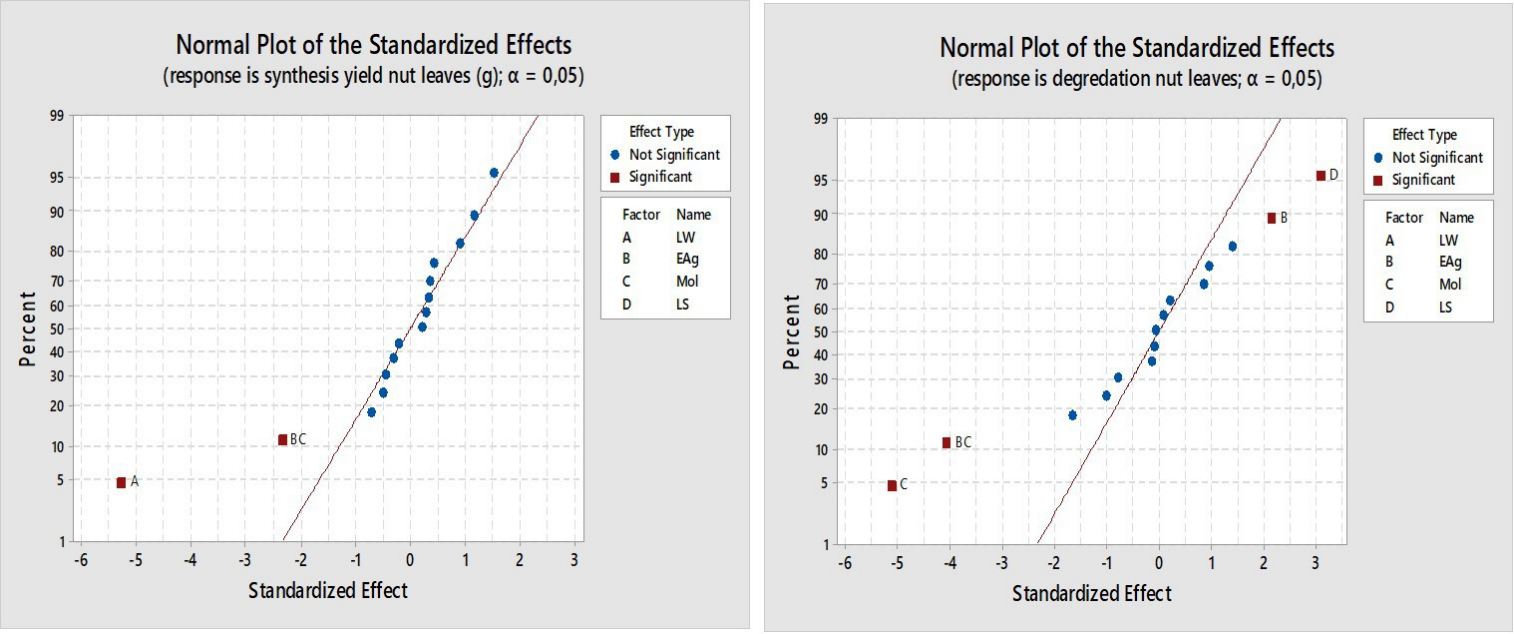
Normal plot of the standardized effects for the responses synthesis yield and degradation.

Figure 4 shows the normal probability plots of the standardized effects for both NP production and dye degradation, providing insights into the relative importance of each factor and their interactions. In the case of NP production, the standardized effects of the LW (A) and the two-way interaction between EAg × Mol (interaction BC) deviate significantly from the reference line and exceed the significance threshold (*α* = 0.05). These results indicate that LW and the EAg × Mol interaction have statistically significant and substantial effects on AgP production, suggesting that both the extraction medium composition and the reaction stoichiometry are key determinants of synthesis yield. In contrast, factors EAg (B), Mol (C), LS (D) and other interaction terms remain close to the straight line, indicating minimal or statistically insignificant effects on particle production.

For dye degradation, the standardized effects of Mol (C), LS (D) and the interaction EAg × Mol (BC) are far off the straight line, indicating that they are important determinants for dye degradation. Among these, Mol exhibits the largest variance identifying Mol as the most influential parameter in dye degradation efficiency. LS and the EAg × Mol interaction also contribute significantly, whereas LW (A) and EAg (B) fall near the reference line, indicating that, on their own, they do not significantly affect dye degradation.

A variety of parameters and their interactions influenced both the synthesis of AgPs and the degradation of MB dye; however, the most significant factors differed between the two processes (table 5). This comparison highlights the differences in the underlying mechanisms governing each process. For AgP synthesis, the water-to-leaf ratio contributed the most (39.43%), indicating that NP yield is strongly influenced by the quantity of water used during extraction. The two-way EAg × Mol interaction contributed 7.78%, suggesting that the NP formation depends not only on individual factor levels but also on their combined effect, particularly in the reduction of silver ions by plant-based phytochemicals. In contrast, dye degradation was primarily governed by Mol, which had the highest contribution (30.38%). This suggests that higher silver ion concentrations promote the formation of more catalytically active NPs. The EAg × Mol interaction again played a key role, contributing 19.09% to the degradation response—more than double its impact on AgP synthesis—highlighting the synergistic importance of extract concentration and silver ion availability in facilitating efficient dye breakdown. LS also showed a notable effect (11.02%), with smaller particles likely enhancing extraction efficiency and thereby increasing the number of active sites for adsorption and catalytic activity. Importantly, the EAg × Mol interaction significantly influenced both responses, but its effect on dye degradation was much more pronounced. This suggests that beyond synthesis, the physicochemical environment created by the interaction of extract and silver ion concentration also determines particle surface properties, dispersion, and reactivity—factors critical for dye removal performance.

**Table 5.** Percentage contributions of significant factors for production and degradation processes.

factor/interaction	particle production (g)	dye degradation (%)
primary factor	LW (39.4%)	Mol (30.38%)
secondary factor	EAg × Mol (7.78%)	EAg × Mol (19.09%)
other factors	non-significant	LS (11.02%)
		EAg (5.33%)

**Figure 5.**
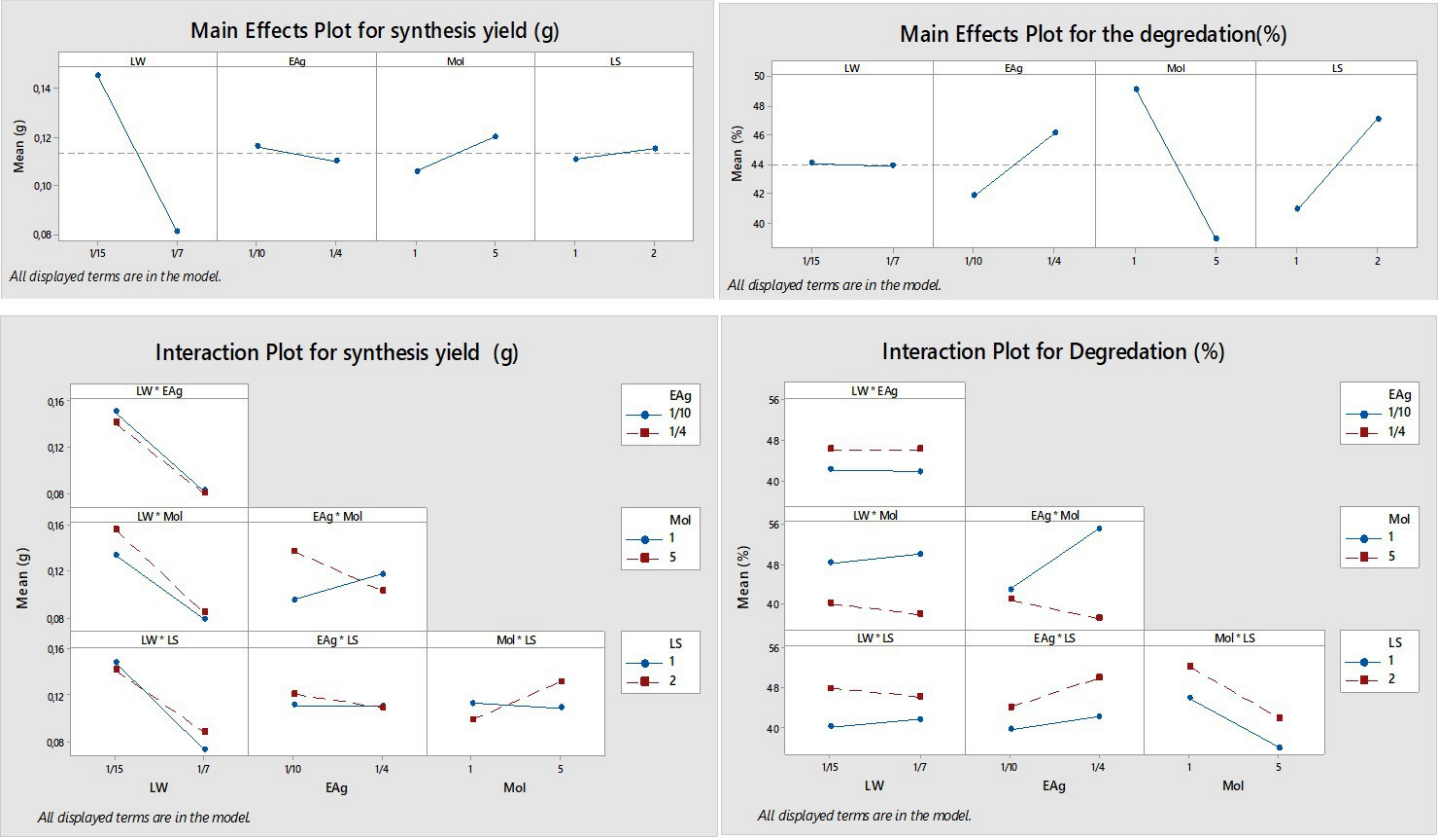
Main effect and interaction plots.

The main effects and the interaction plots are given in figure 5. For AgP synthesis, nucleation and particle growth are thought to be the main mechanism, where regulation of the silver ion reduction and LW play a crucial role. Since the LW directly affects the concentration of phytochemicals responsible for reducing silver ions, its optimization is essential for maximizing NP yield. The plot shows a clear negative slope for LW, indicating that increasing the LW from 1:15 to 1:7 significantly reduces NP production. A lower LW (1:15) results in a more concentrated extract, thereby enhancing the availability of reducing agents and promoting particle formation. Although the EAg has an effect, its effect is not as strong as LW’s. Higher extract ratios may enhance the reduction of silver ions, but the trend is not as distinct. Similarly, increasing Mol provides more silver ions for particle formation; however, excessively high concentrations may promote aggregation, reducing synthesis efficiency. LS appears to have a minimal effect, as both small and large sizes seem comparably effective in releasing bioactive compounds required for reduction. The interaction of EAg × Mol is statistically significant as evidenced by the non-parallel lines in the interaction plot. This indicates that the effect of extract concentration on NP production is dependent on the level of Mol. Therefore, optimizing both the extract-to-silver ratio and silver ion concentration is crucial to achieving maximum NP yield.

The main effects and interaction plots reveal that the EAg has a positive effect on dye degradation, while Mol exhibits a negative impact. A noticeable upward trend in the EAg plot indicates that increasing the ratio from 1:10 to 1:4 enhances dye removal efficiency. In contrast, increasing the Mol from 1 mM to 5 mM is associated with a sharp decline in dye degradation efficiency. This negative slope suggests that higher silver ion concentrations may lead to the formation of larger or aggregated particles with reduced surface area and catalytic activity, thereby impairing dye removal performance. A positive trend is also observed with LS, where reducing the size from level 2 to level 1 improves dye degradation, possibly due to more efficient extraction of bioactive compounds from smaller leaf fragments. The interaction between Mol and EAg is statistically significant. At low Mol (1 mM), increasing EAg from 1:10 to 1:4 slightly enhances dye degradation. Although the improvement is less pronounced at high Mol (5 mM), the interaction effect remains important. This suggests that extract concentration is a more dominant factor when silver ion availability is limited and that optimizing both variables is necessary for achieving maximum dye removal efficiency.


(3.1) $$\begin{array}{r} NP\;Produced\;(gr)=0.11327-0.03203\;LW-0.00303\;EAg+0.00709\;Mol+0.00215\;LS+0.00169\;LW\;\times EAg-0.00441\;LW\times Mol+0.00548\;LW\times LS-0.01423\;EAg\times Mol-0.00280\;EAg\times LS+0.00921\;\;Mol\times LS+0.00255\;LW\times EAg\times Mol+0.00194\;LW\times EAg\times LS+0.00128\;LW\times Mol\times LS-0.00196\;\;EAg\;\times Mol\times LS-0.00127\;LW\times EAg\times Mol\times LS \end{array}$$

(3.2) $$\begin{array}{r} Degradation\;efficiency\;(1\;hour)(\%)=44.011-0.095\;LW+2.134EAg-5.097\;Mol+3.070\;LS+\;0.078\;LW\times \;EAg-1.016\;LW\times \;Mol-0.795\;LW\times \;LS-4.041\;EAg\times \;Mol+0.843\;EAg\times LS-0.147\;\;Mol\times \;LS-0.059\;LW\times \;EAg\times \;Mol+0.211\;LW\times \;EAg\times \;LS+1.376\;LW\times \;Mol\times \;LS-\;1.657EAg\;\times Mol\times LS+0.949LW\times EAg\times Mol\times LS \end{array}$$

The results of the optimization analysis are given in table 6. The factors LW and LS were found to be identical for both response variables, indicating a shared optimal range for these parameters.

**Table 6.** Optimum AgP synthesis conditions.

responses	leaf:water (LW)	extract:AgNO_3_ (EAg)	AgNO_3_ molarity (Mol)	**leaf size** (LS)	maximum yield
NP production (g)	1:15	1:10	5	2	0.131
MB degradation (%)	1:15	1:4	1	2	62.85

The optimal conditions include an EAg of 1:10 and Mol of 5 mM, suggesting that a higher availability of silver ions and a lower concentration of reducing agents promote greater NP yield. Conversely, to maximize MB degradation efficiency, the optimal conditions shift to an EAg of 1:4 and a Mol of 1 mM. These conditions favour the formation of smaller, more catalytically active particles, likely due to reduced aggregation and higher surface area, thereby enhancing dye removal performance.

Silver NPs have been synthesized and characterized in numerous studies employing various green sources and optimization strategies. For instance, Kartini *et al.* investigated the use of three different plant extracts—PN, OS, and CL—at five different concentrations (0.125−1%) to produce silver NPs. Among the tested extracts, PN at 0.5% concentration yielded NPs with the most favourable characteristics and highest yield [10]. Similarly, in the present study, the EAg was found to play a critical role, particularly in combination with Mol. The interaction of these parameters significantly affected both NP synthesis and dye degradation, emphasizing the importance of optimizing both individual factors and their interactions. In another study, the green production of silver NPs was optimized using response surface methodology (RSM) with FFD. The authors reported that the concentration of sodium hydroxide (NaOH) was the most influential factor, followed by AgNO₃ and extract concentrations [11]. Likewise, using BPE, silver NPs were synthesized, and RSM was used to optimize the synthesis conditions through CCD. Four independent parameters were selected: pH level (2.3−10.1), incubation period (24−120 hours) and concentrations of silver nitrate (AgNO_3_) and BPE (0.25−2.25 mM, 0.2–1.96% (v/v) correspondingly). BPE concentration of 1.7% (v/v), AgNO_3_ concentration of 1.75 mM, 48 hour incubation duration and pH level of 4.3 were found to be the ideal combination of all independent parameters, resulting in the shortest peak wavelength and peak width [12]. Optimal NP synthesis has often been reported under moderate conditions of both extract and metal salt concentrations, as well as slightly acidic pH levels. Consistent with these findings, our study also demonstrates that mid-range levels of EAg and Mol lead to improved dye degradation performance, likely due to the balanced formation of smaller, more active particles and minimized aggregation. For example, Barati *et al.* [13] employed RSM with a face-centred CCD to synthesize CuO NPs using walnut green husk aqueous extract. Their optimization focused on three critical factors: reaction time, extract volume and copper acetate concentration. The optimal synthesis resulted in amorphous, monodisperse particles with an average hydrodynamic diameter of 82.61 nm, achieved with a 1 mM salt concentration, 1 ml extract per 10 ml of reaction volume and a 1 hour reaction time [13]. These factors are analogous to LS, EAg and Mol in our study, supporting the idea that careful tuning of plant-based synthesis variables can control NP characteristics such as surface area, size and dispersity, all of which influence catalytic activity.

These results emphasize the value of employing factorial experimental design in the green synthesis of AgPs. This approach not only enables the efficient identification and ranking of influential synthesis parameters but it also helps identify synergistic interactions that might otherwise be overlooked. The statistical evaluation clearly demonstrated how synthesis variables such as the EAg ratio and Mol interact synergistically to affect NP yield and functionality. Furthermore, the high pseudo-second-order (PSO) rate constant (*k*₂ = 67 × 10⁻³ mg g⁻¹ min⁻¹) and the consistency with Langmuir–Hinshelwood (L–H) kinetics highlight the dual role of both adsorptive and catalytic processes, directly influenced by particle surface characteristics shaped by synthesis parameters.

### 3.2. Characterization of the particles

**Figure 6.**
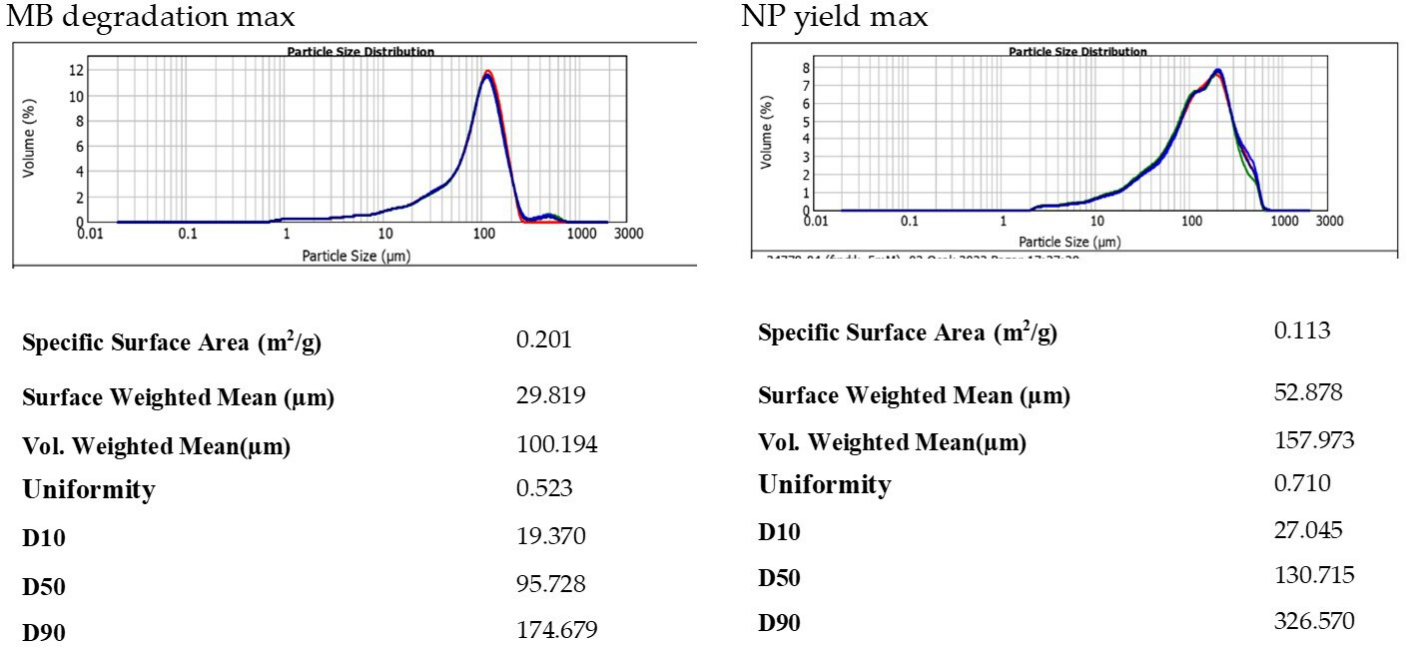
Particle size distribution of the particles for the maximum of the two responses NP production and MB degradation.

Figure 6 presents the results of the particle size analysis. The data reveal that AgPs synthesized under conditions optimized for MB degradation exhibited smaller particle sizes and higher total surface areas compared to those optimized for maximum NP yield. Specifically, the surface-weighted mean diameter for the MB degradation condition (29.819 µm) was 1.77 times lower than that observed for the NP yield condition (52.878 µm). This indicates a greater proportion of fine particles, which enhances the catalytic surface area available for dye interaction. Additionally, the narrower particle size distribution, as reflected in the lower D10, D50 and D90 values, further supports the enhanced homogeneity and surface efficiency of particles in the MB degradation condition. In contrast, broader and larger distributions observed under high-yield conditions suggest aggregation or growth-dominant synthesis.

**Figure 7.**
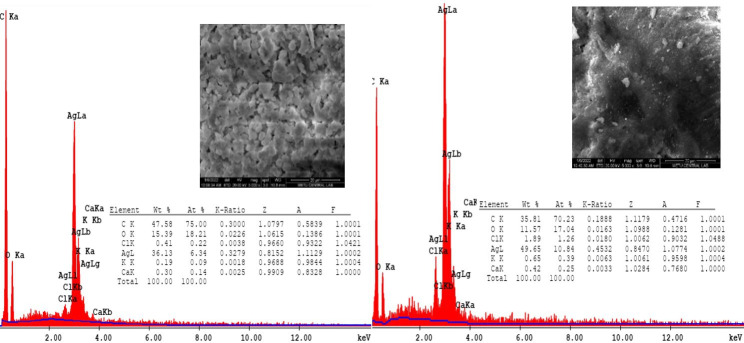
SEM-EDX of the produced particles: (a) NP synthesis and (b) MB degradation.

Figure 7 illustrates SEM and EDX mapping results, highlighting surface morphology and elemental composition of the AgPs. EDX results of the particles show that the percentages of C and O were higher under the MB degradation condition, suggesting that there may be more functional groups on the surface. Conversely, samples from maximum yield conditions displayed higher silver (Ag) content, indicating a more metallic and stable particle structure, suitable for long-term storage but less active in catalytic processes.

**Figure 8.**
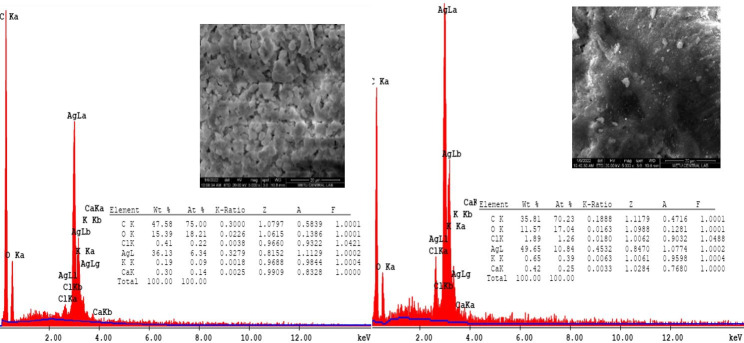
Degradation mechanism of MB dye with AgPs.

Monitoring the change in the contaminants’ concentration over time is the primary method used to assess heterogeneous photocatalytic degradation processes, which convert soluble organic contaminants into CO_2_ and H_2_O. Figure 8 illustrates the proposed mechanism for MB degradation using AgPs as a catalyst under solar radiation. This figure shows that the photocatalytic activity of AgPs arises from two key mechanisms: the inter-band transition and the localized surface plasmon resonance (LSPR) characteristic. The LSPR occurs when the frequency of the incident wave and the collective oscillation frequency of the NP electrons, which excite the electrons and raise their energy state, match. Due to their high energy or high temperature, these electrons can provide enough energy to make oxygen free radicals (O_2_•), which then react with the hydroxyl ions and oxygen (O_2_) molecules in the environment to form hydroxyl radicals (OH•). The dye molecules may be attacked by these free radicals, leading to their degradation [15,16].

To determine photodegradation kinetic parameters, the most widely adopted method is to employ a linearized version of the PFO and PSO models [17]. Numerous studies have been conducted on the kinetics of MB degradation utilizing silver NPs, which have revealed multiple aspects of the catalytic process. These studies have demonstrated that the reaction kinetics can vary significantly depending on experimental conditions. For instance, the presence of oxidizing agents or changes in light intensity may shift the reaction order, resulting in zero-, first-, or second-order behaviour under different circumstances [18].

The linear form of the PFO and the linear form of the PSO kinetic model are shown in equations (3.3) and (3.4):


(3.3) $$\ln(q_{e}-q_{t})=\ln q_{e}-k_{1}t,$$

(3.4) $$\frac{t}{q_{t}}=\frac{1}{k_{2}q_{e}^{2}}+\frac{1}{q_{e}},$$

where *k*_1_ is the PFO rate constant, *k*_2_ is the PSO rate constant, *q*_e_ is the amount of dye adsorbed at equilibrium per unit weight of the adsorbent (mg g^−1^ min^−1^) and *q*_t_ is the amount of solute adsorbed (mg g^−1^ min^−1^) at time *t* (minute).

One of the most widely used models for describing the kinetics of heterogeneous catalytic systems is the L–H model. The following equation is used to analyse the L–H model [19,20]:


(3.5) $$\ln\frac{c_{0}}{c_{t}}=k_{app}\times t,$$

where *c*_0_ is the initial concentration (mg l^−1^), *c*_t_ is the concentration at a time *t* (minute) and *k*_app_ is the apparent reaction constant.

**Figure 9.**
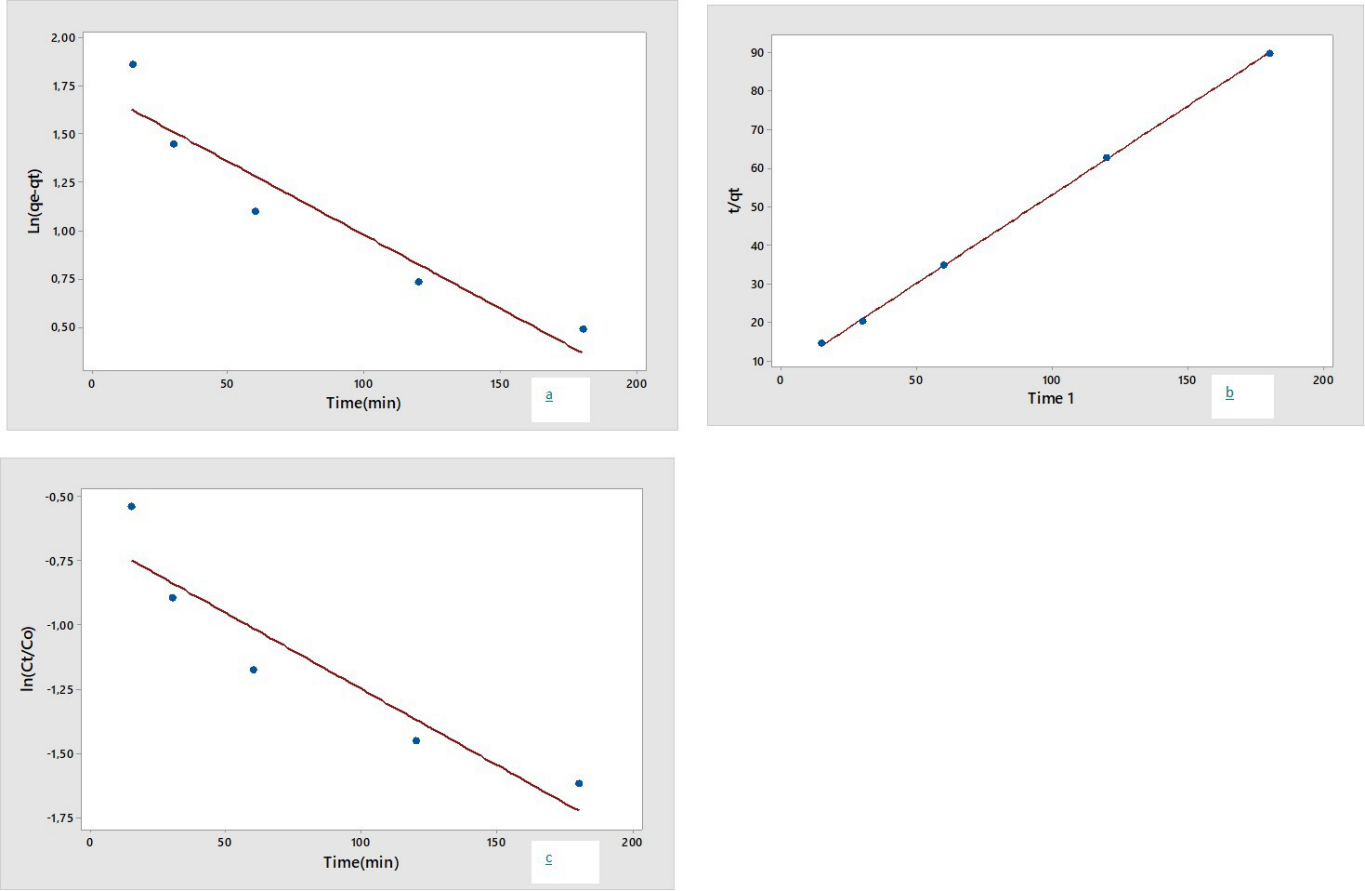
Reaction kinetics of the photodegradation: (a) pseudo-first-order, (b) pseudo-second-order and (c) Langmuir–Hinshelwood kinetics.

The kinetic model fits are given in figure 9, and the corresponding kinetic coefficients are summarized in table 7. Among the tested models, the PSO kinetic model provided the best fit to the experimental data, with a correlation coefficient (*R*²) exceeding 0.99. This strong agreement suggests that the rate-limiting step of the MB degradation process is chemisorption, involving valency forces through sharing or exchange of electrons between dye molecules and active sites on the NP surface. In contrast, the L–H model showed a lower correlation (*R*² = 0.88), indicating that although surface reactions may contribute to the overall degradation mechanism, their role is less dominant compared to the adsorption-controlled pathway. This finding emphasizes that the green-synthesized AgPs primarily act as adsorptive catalysts, with limited contribution from surface-bound reactive species in the dye removal process.

**Table 7.** Comparison of kinetic coefficients and degradation percentage of MB for different studies.

*c*_0_ (mg l^−1^)	particle conc.	degradation (%)	kinetic coefficients	ref.
0.5	0.2 g AgPs l^−1^	62.85	*k*_1_: 0.042 × 10^–3^ min^–1^ *R*^2^: 0.90 *k*_2_: 67 × 10^–3^ mg g^–1^ min^–1^ *R*^2^: 0.99 *k*_app_: 5.9 min^–1^ *R*^2^: 0.88	this study
100	0.05−0.5 g CuO l^−1^	98.89	*k*_1_: 0.62 × 10^–3^ min^–1^ *R*^2^: 0.97 *k*_2_: 56.42 × 10^–3^ mg g^–1^ min^–1^ *R*^2^: 0.97 *K*_id_: 0.843 mg g^–1^ min^1/2^ *R*^2^: 0.98	[21]
10	1 g ZnFe_2_O_4_		*k*_1_: 1.3 × 10^–3^ min^–1^ *R*^2^: 0.13 *k*_2_: 110 × 10^–3^ mg g^–1^ min^–1^ *R*^2^: 0.99 *K*_id_: 0.62 mg g^–1^ min^1/2^ *R*^2^: 0.78	[22]
10	4 g ZnO l^−1^	88.05	*k*_1_: 14.77 × 10^–3^ min^–1^ *R*^2^: 0.78 *k*_2_: 75 × 10^–3^ mg g^–1^ min^–1^	[23]
7.4	2.25 g FeO l^−1^		*k*_1_: 13 × 10^–3^ min^–1^ *R*^2^: 0.97 *k*_2_: 1.2 × 10^–3^ mg g^–1^ min^–1^ *R*^2^: 0.98	
3.2	2.5 g ZnO l^−1^	99.86	*k*_app_ : 21 min^-1^ *R*^2^: 0.88	[14]
60	1800 ml AgPs l^−1^		*k*_1_: 11 × 10^–3^ min^–1^ *R*^2^: 0.96 *k*_2_: 5.7 l g^–1^ min^–1^ *R*^2^: 0.98	[24]
1	0.1g AgPs l^−1^	97.57	*k*_1_: 23 × 10^–3^ min^–1^ *R*^2^: 0.89 *k*_2_: 5.7 l g^–1^ min^–1^ *R*^2^: 0.98	[16]

The comparison of this study with existing literature highlights the critical role of NP type, synthesis conditions and experimental parameters in dye degradation efficiency. In the present study, biogenically synthesized AgPs achieved a moderate degradation efficiency of 62.85% under relatively low catalyst concentration and initial dye concentration. Despite the moderate removal rate, the reaction kinetics were best described by the PSO model with a high correlation coefficient (*R*² = 0.99), indicating that chemisorption—characterized by electron exchange or covalent bonding between the dye and catalyst surface—was the dominant mechanism. This finding aligns with previous reports in the literature, which similarly identified PSO kinetics as the prevailing model across a variety of NP systems, including metal oxides and plant-mediated metallic NPs.

In comparison, studies involving CuO- and ZnO-based NPs have reported higher degradation efficiencies, particularly under conditions of increased NP dosage and higher initial dye concentrations [10–12]. These variations can be attributed to metal oxides’ inherent photocatalytic qualities, increased surface reactivity and oxygen vacancies. Even at lower reactant concentrations, the kinetic performance (rate constants and *R*^2^ values) of the AgPs synthesized in this work was shown to be equivalent or better, highlighting the importance of surface characteristics over degradation rate alone. This aligns with the trend observed in other studies where PSO kinetics dominate, indicating chemisorption as the primary mechanism. Comparatively, CuO- and ZnO-based NPs demonstrated higher degradation efficiencies, particularly at higher particle concentrations and initial dye concentrations, reflecting their enhanced reactivity and adsorption capacity. Moreover, in our study, the L–H kinetic model also showed a reasonable fit (*k*_app_ = 5.9 min⁻¹, *R*² = 0.88), indicating the contribution of surface-mediated reactions to the overall mechanism. This dual kinetic behaviour supports the hypothesis that both adsorption and catalytic surface reactions are involved. Additionally, particle size and surface area analyses further support these findings: AgPs synthesized under optimized dye degradation conditions exhibited smaller particle sizes and larger surface areas (0.201 m² g^−1^) compared to those optimized for maximum synthesis yield (0.113 m² g^−1^). This reinforces the idea that smaller NPs with greater surface area and functional group availability play a critical role in enhancing catalytic activity.

## 4. Conclusion

This study successfully demonstrated the optimization of biogenic AgP synthesis for MB degradation through a FFD approach. By systematically evaluating four key process parameters—LW, EAg, Mol and LS—the study identified the most influential factors affecting both NP yield and dye degradation efficiency.

The results showed that LW and the EAg × Mol interaction significantly impacted AgP production, whereas Mol, LS and the interaction term EAg × Mol were the primary contributors to degradation. Kinetic modelling confirmed that the degradation process followed a PSO mechanism, indicating chemisorption, and the L–H model further supported the involvement of surface-mediated catalytic reactions.

Characterization results further confirmed that AgPs synthesized under optimal dye degradation conditions had smaller particle sizes and higher specific surface areas, enhancing their catalytic performance. These findings underscore the importance of tailoring synthesis parameters according to the desired application—whether prioritizing NP yield or maximizing functional efficiency in dye degradation. Overall, the study highlights the effectiveness of statistical design of experiments in optimizing green synthesis processes and demonstrates the potential of biogenically synthesized AgPs as eco-friendly and efficient catalysts for wastewater treatment and other environmental remediation applications.

## Data Availability

All data supporting the findings of this study are available in the Figshare repository [25].
